# Menin Modulates Mammary Epithelial Cell Numbers in Bovine Mammary Glands Through *Cyclin D1*

**DOI:** 10.1007/s10911-017-9385-8

**Published:** 2017-11-29

**Authors:** Kerong Shi, Xue Liu, Honghui Li, Xueyan Lin, Zhengui Yan, Qiaoqiao Cao, Meng Zhao, Zhongjin Xu, Zhonghua Wang

**Affiliations:** 1 College of Animal Science and Technology, Shandong Agricultural University, Taian, 271018 China; 2 Shandong Key Laboratory of Animal Bioengineering and Disease Prevention, Taian, 271018 China

**Keywords:** *MEN1*/menin, Mammary epithelial cells, Cell growth, Cyclin D1

## Abstract

Menin, the protein encoded by the *MEN1* gene, is abundantly expressed in the epithelial cells of mammary glands. Here, we found *MEN1*/menin expression slowly decreased with advancing lactation but increased by the end of lactation. It happened that the number of bovine mammary epithelial cells decreases since lactation, suggesting a role of menin in the control of mammary epithelial cell growth. Indeed, reduction of menin expression through *MEN1*-specific siRNA transfection in the bovine mammary epithelial cells caused cell growth arrest in G1/S phase. Decreased mRNA and protein expression of Cyclin D1 was observed upon *MEN1* knockdown. Furthermore, menin was confirmed to physically bind to the promoter region of C*yclin D1* through a ChIP assay, indicating that menin plays a regulatory role in mammary epithelial cell cycle progression. Moreover, lower expression of *MEN1*/menin induced increased epithelial cell apoptosis and caused extracellular matrix remodeling by down-regulating its associated genes, such as *DSG2* and *KRT5*, suggesting that menin’s role may also be involved in the control of cell–cell adhesion in normal mammary glands. Taken together, our data revealed an unknown molecular function of menin in epithelial cell proliferation, which may be important in the regulation of lactation behavior of mammary glands.

**Electronic supplementary material:**

The online version of this article (10.1007/s10911-017-9385-8) contains supplementary material, which is available to authorized users.

## Introduction

Menin, a protein of 610 amino acids encoded by the *MEN1* gene, plays an important regulatory role in the metabolism of organisms. Mutations in *MEN1* gene and therefore mutant protein menin predispose patients to multiple endocrine neoplasia type 1 (MEN1) syndrome, which is characterized by the occurrence of multiple endocrine tumors, mainly affecting the parathyroid glands, pituitary (anterior lobe) and pancreatic islets [1, 3−3]. These endocrine organs secrete hormones such as prolactin and insulin, which can physiologically modulate the lactation curve pattern of mammary glands. Moreover, prolactin and/or insulin could be downstream factors of menin, which regulates their gene transcription [4, 6−6]. This suggests the existence of a link between *MEN1*/menin and hormone-dependent tissues [7, 9−9].

Within the mammary gland, menin was recently found to directly interact with estrogen receptor-α (ERα) in a hormone-dependent manner in breast cancer cells [10], thereby regulating the transcription of estrogen-responsive genes in these cells [11]. This finding indicated an important mediator role of menin in mammary gland tissue of dairy cows [12]. Moreover, loss of heterozygosity of *MEN1* was found to cause inherited breast-ovarian cancer in humans [13] and hyperplasia of breast (female) and prostate (male) cells in mice [9, 14]. These findings indicated a possible regulatory role of *MEN1*/menin in cell growth and/or cell cycle progression in mammary gland tissue.

The milk yield and shape of the lactation curve of a dairy cow are determined by the number of mammary epithelial cells and their secretory activity. Nearly all of the cells present in the bovine mammary gland are formed by calving [15]. During early lactation, the number of mammary epithelial cells within the mammary glands is at a peak and then gradually decreases with advancing lactation [15]. The decline in mammary cell numbers during lactation must at least partially account for the decline in milk yield after the peak stage, which occurs approximately 60–90 days after parturition [16]. Accompanying the decline in mammary cell numbers, apoptotic cell death and a degree of cell renewal and lobular-alveolar remodeling are observed [17, 18]. A change in the rate of the decline in cell numbers and/or apoptosis could provide a means of modulating milk yield and mammary gland lactation curve pattern. However, the molecular mechanisms controlling mammary cell growth/survival are poorly defined. It was hypothesized that there were important inherited genetic factors regulating the epithelial cell growth and/or death, therefore making effects on the lactation persistency and milk yield of mammary glands. Hence, the objective of the present investigation was to assess the molecular mechanisms of *MEN1*/menin in regulating epithelial cell growth in bovine mammary glands. A bovine mammary epithelial cell line, MAC-T, was used as the experimental model [19, 21−21], in addition to mammary gland tissues at different lactation stages.

## Results

### *MEN1*/menin Knowdown in Mammary Epithelial Cells

The expression level of *MEN1* and it encoded protein menin were investigated during five different lactation stages (including the dry period) in mammary glands of dairy cows, the results indicated both *MEN1* mRNA and the menin protein slowly decreased with advancing lactation, but later increased after the peak milk stage through the dry period (or involution stage), with the lowest expression level being observed around the peak lactation stage (55 ± 4.3 days in milk, DIM; supplementary Fig. S1).

**Fig. 1:**
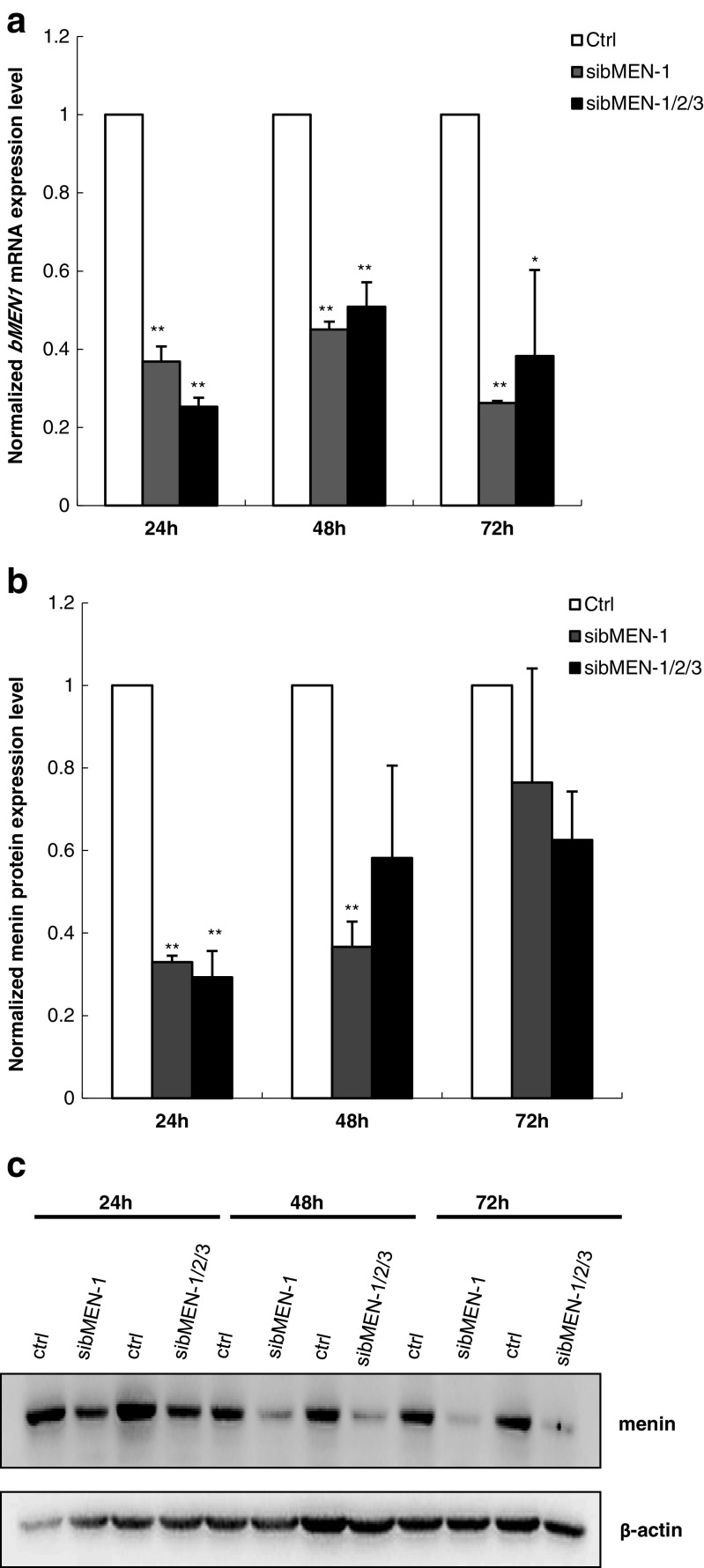
***MEN1***/**menin expression was successfully knocked down in mammary epithelial cells**. MAC-T cells were transfected with single *MEN1*-specific siRNA(sibMEN-1) or pooled siRNAs (sibMEN-1/2/3). The expression levels of *MEN1* mRNA (**a**) and menin protein (**b**) were determined via qRT-PCR and western blott at 24, 48 and 72 h after transfection based on three times of replicate experiments. The data are shown as normalized expression levels to the internal control β-actin, compared with their corresponding controls. *P < 0.05, ** P < 0.01. (**c**) Representative western blot images of the expression of menin and the loading control β-actin are shown. Cells at 24 h after transfection with the *MEN1*-specific siRNA pool were ultimately chosen for subsequent analyses, because of its lowest *MEN1* gene and also protein expression in time course studies

To assess the possible regulatory function of the *MEN1* gene in the mammary glands of dairy cows, *MEN1*/menin expression levels were modified in vitro in bovine mammary epithelial cells (MAC-T cells) to mimic the decrease in expression after the initiation of lactation in the mammary gland. In vitro-synthesized siRNAs specific to the bovine *MEN1* gene were transfected either separately (sibMEN1-1, sibMEN1-2 or sibMEN1-3) or in combination (sibMEN1-1/2/3, as a siRNA pool) into MAC-T cells (data not shown). sibMEN1-1/2/3 transfection were shown to result in the best knockdown efficiency of *MEN1* and menin at 24 h posttransfection via qRT-PCR and western blot detection, respectively. The siRNA pool (sibMEN1-1/2/3) was ultimately used for the following experiments because of the best knockdown level (25.31%±3.86% of the *MEN1* mRNA level, Fig. 1a; and 29.26%±11.10% of the menin protein level in untransfected MAC-T cells, respectively; Fig. 1b, c).

### Knockdown of *MEN1*/menin Induces Extracellular Matrix Remodeling

**Fig. 2:**
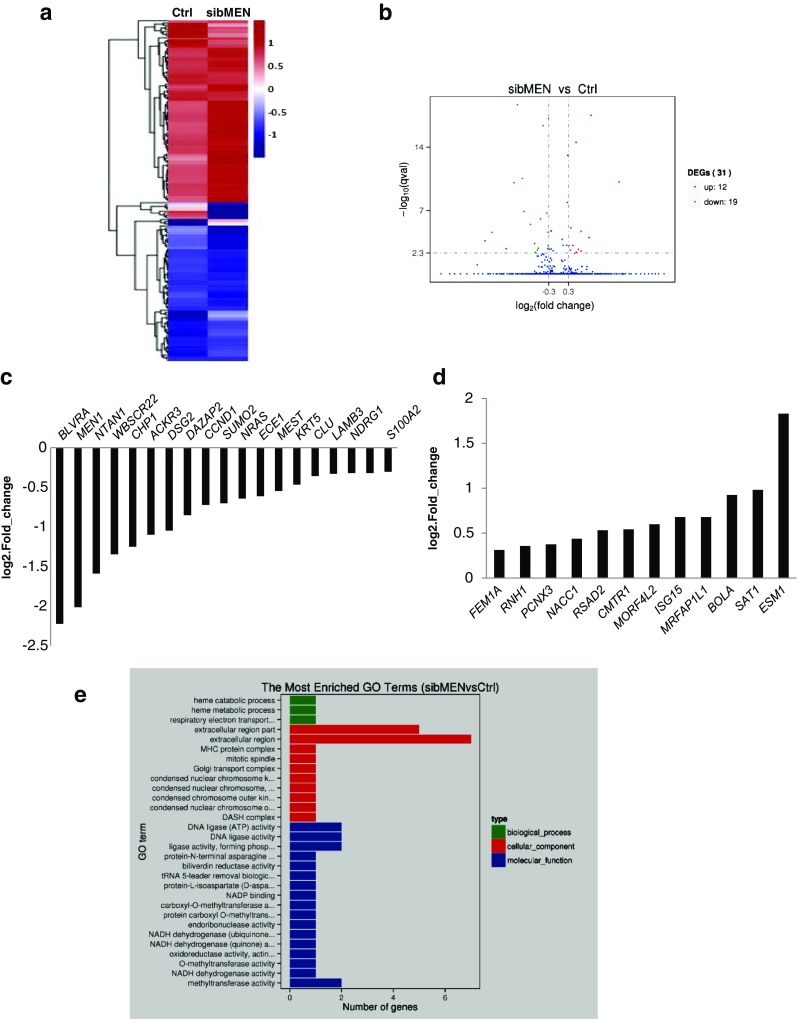
**The differentially expressed genes upon** ***MEN1***/**menin knockdown in MAC-T cells were enriched into extracellular matrix remodeling activity**. Three independent replicates of MAC-T cells that transiently transfected with control siRNA (Ctrl) and *MEN1*-specific siRNAs (sibMEN) were separately collected for total RNA extraction, followed by the evaluation of the efficiency of *MEN1* knockdown (79.0±0.23%), were then pooled with equal proportions for RNAseq analysis. **a** Heat map clusters showing differentially expressed genes upon *MEN1*/menin knockdown in MAC-T cells. The different colors in the scale bar indicate the fold-changes of gene expression. **b** Volcano plot of differentially expressed genes. The numbers of up- and down-regulated genes are indicated on the right. **c** The 19 down-regulated genes (such as *DSG2, KRT5, BLVRA* and *CCND1*) detected upon *MEN1*/menin knockdown in MAC-T cells are shown. The *MEN1* gene was included in the gene list as a positive control. **d** The 12 up-regulated genes detected upon *MEN1*/menin knockdown in MAC-T cells are shown. **e** Gene Ontology (GO) analysis of the differentially expressed genes (*KRT5, LAMB3, MEST, DSG2*, etc.) indicated that extracellular matrix remodeling was the most enhanced activity upon the reduction of *MEN1*/menin in MAC-T cells. The colors present three different ontology domains; the x-directed bars present the enriched GO terms

Three independent replicates with lower expression of *MEN1*/menin were pooled and subjected to high-throughput sequencing (Fig. 2a). These samples exhibited 21.04%±0.24% (n = 3) of *MEN1* mRNA and 22.87%±1.30% (n = 3) of the menin protein level found in untransfected MAC-T cells. The results showed 31 genes presented differential expression upon *MEN1* knockdown (Fig. 2b), with 19 genes being down-regulated (Fig. 2c) and 12 being up-regulated (Fig. 2d). GO analysis indicated that most down-regulated genes were mainly enriched into extracellular matrix remodeling process upon *MEN1* knockdown in mammary epithelial cells (Fig. 2e).

### Knockdown of *MEN1*/menin Arrests Mammary Epithelial Cells in G1/G0 Phase and Delays the G1/S Phase Transition

**Fig. 3:**
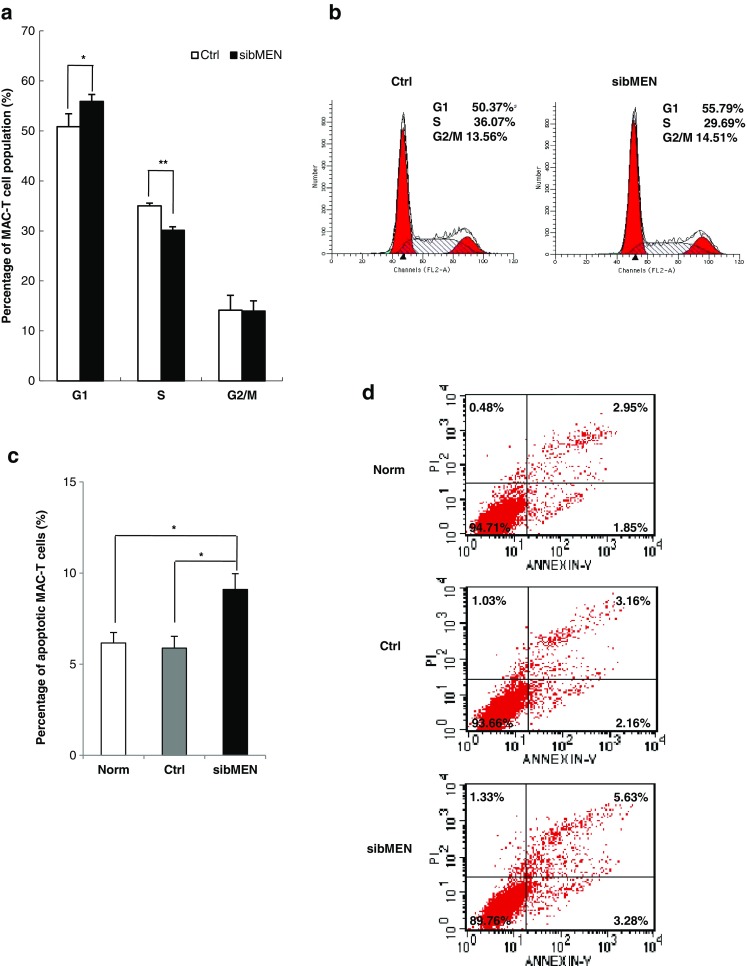
***MEN1***/**menin reduction caused mammary epithelial cell growth arrest at G1**/**S phase. a** MAC-T cells were exposed to the indicated siRNAs for 24 h and assessed for the distribution of cell cycle phases after propidium idodide (PI) staining. The percentages of cells in G0/G1, S, and G2/M phases are shown. **P < 0.01 and *P < 0.05. **b** Representative FACS images of cell phase analyses illustrating changes in the cell cycle in MAC-T cells upon *MEN1*/menin knockdown compared with the negative control. The cell cycle phase distribution (%) is indicated within each panel. **c** The same treated cells were also assessed for the distribution of apoptotic status after annexin V-FITC/propidium iodide (PI) staining. “Norm” represents the plain cells without any treatments; “Ctrl” and “sibMEN” represent the cells treated with scramble and *MEN1*-specific siRNAs, respectively. **P < 0.01 and *P < 0.05. **d** Representative images of apoptotic analyses illustrating the percentage of apoptotic MAC-T cells upon *MEN1*/menin treatment. The percentages of cells in different apoptotic stages are indicated within each panel

Upon *MEN1* knockdown in mammary epithelial cells, propidium iodide (PI) staining was performed in cells for analysis of the cell phase distribution via flow cytometry, along with the corresponding control cells. The results showed that decreased *MEN1* expression substantially increased the percentage of cells by 5.07% in G1/G0 phase (P = 0.026) and decreased the percentage of cells by 4.88% in S phase (P = 0.0019; Fig. 3a, b). To further examine the potential role of menin in controlling epithelial cell growth, we performed immunofluorescence staining of exponentially growing menin-knockdown MAC-T cells using annexin V-FITC and found that the number of annexin V-positive cells was increased in cells with lower menin expression (Fig. 3c, d), indicating cell apoptosis (supplementary Fig. S4). These results suggested that menin suppression causes inhibition of the G1/S transition of bovine mammary epithelial cells.

### Menin Down-Regulates of Cyclin D1 Expression

**Fig. 4:**
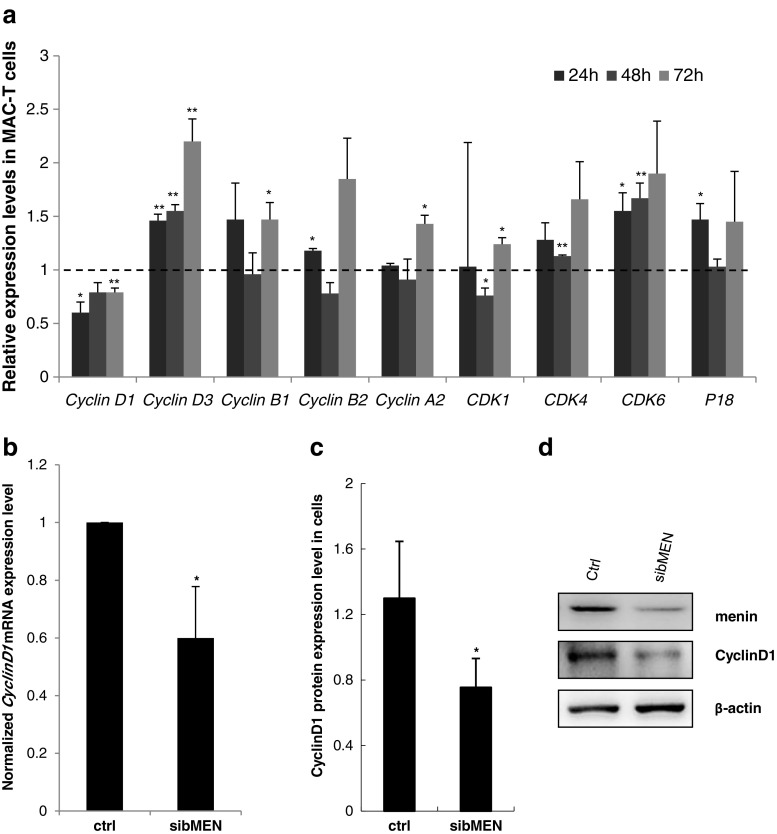
***CycinD1*** **was significantly suppressed upon** ***MEN1***/**menin knockdown. a** The expression of cell cycle progression associated genes was assessed in the MAC-T cells after 24, 48 and 72 h of transfection with *MEN1*-specific siRNAs, as well as their negative controls. The dotted line represents the expression levels of each genes in their negative control cells at the indicated time points. *Cyclin D1* expression was significantly inhibited all time points. **P < 0.01, *P < 0.05. **b** The suppression of *Cyclin D1* mRNA expression was confirmed by three independent *MEN1* knockdown experiments at 24 h posttransfection. The data are shown as relative expression levels normalized to the internal control β-actin. *P < 0.05. **c** Cyclin D1 protein expression was suppressed upon *MEN1*/menin knockdown (n = 3). *P < 0.05. **d** Representative western blot images of the expression of menin, Cyclin D1 and the loading control β-actin are shown

Since the suppression of *MEN1*/menin expression results in cell growth arrest in the G1/S phase, it was logical to question whether cell cycle regulatory genes are modulated by menin. Hence, the expression of candidate genes that might specifically control the G1/S transition was detected in MAC-T cells upon *MEN1* knockdown (Fig. 3a, Supplementary Table S2). The time course *MEN1* knockdown induced that the mRNA expression of *Cyclin D1* significantly decreased (P < 0.05 for all the three time points), whereas those of CDK4 (P = 0.009 at 48 h), *CDK6* (P = 0.03 at 24 h and P = 0.01 at 48 h) and *p18* (a G1/S phase-specific cell cycle inhibitor; P = 0.04 at 24 h) increased (Fig. 4a) in various degree upon *MEN1*/menin knockdown, compared to their corresponding negative controls. At 24 h post-transfection, the expression of *Cyclin D1* was further confirmed to be significantly down-regulated in low menin-expressing mammary gland epithelial cells at both the mRNA (Fig. 4b, P = 0.03) and protein (Fig. 4c, d, P = 0.04) levels. These findings were validated by high-throughput sequencing data from epithelial cells, and *Cyclin D1* (also named as *CCND1*) was one of the down-regulated genes identified upon *MEN1* knockdown (Fig. 2c).

### Menin Associates with the *Cyclin D1* Promoter

**Fig. 5:**
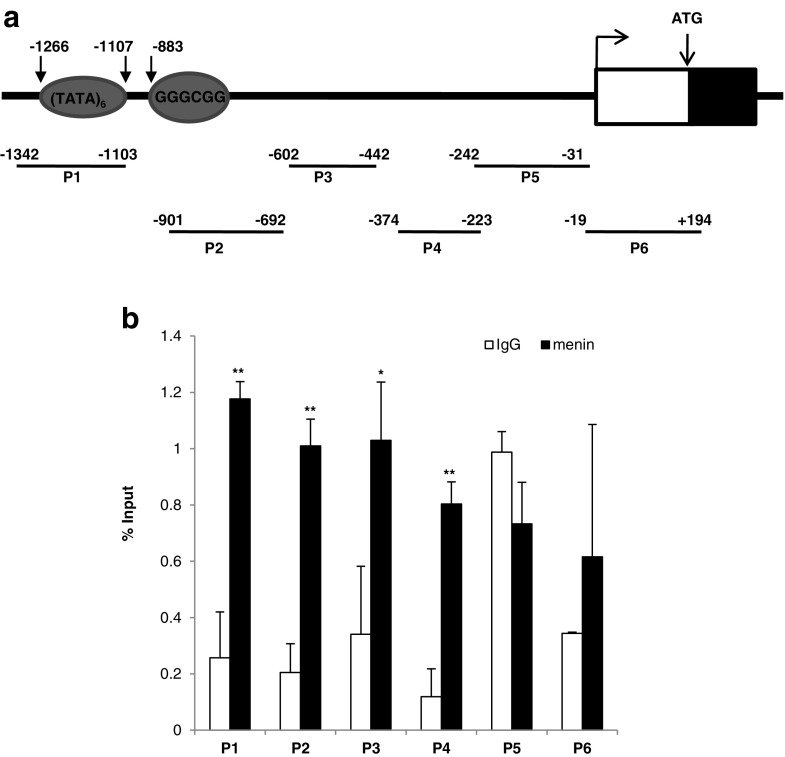
***Cyclin D1*** **is a transcriptional target of menin. a** Six amplicons (P1, P2, P3, P4, P5 and P6) were used to detect the indicated regions of the *Cyclin D1* promoter for menin-ChIP assays (n = 3). The potential promoter elements to which RNA polymerase II (Pol II) usually binds are shown in grey-filled ovals. There were up to six TATA-alike boxes and one CCAAT box (not shown) in the P1 region, and a classical GC box with the conserved sequence GGGCGG was present in the P2 region. **b** Results of quantitative menin-ChIP PCR assays in MAC-T cells are shown, as is the negative control IgG-ChIP PCR. The results were representative of three independent ChIP experiments, showing as the percentage of input by quantifying the amount of chromatin obtained from immunoprecipitation relative to the amount in the input samples. **P < 0.01, *P < 0.05

We hypothesized that menin might associate with promoter regions of *Cyclin D1* and repress the transcription of *Cyclin D1*, obstructing the recruitment of other transcriptional regulators. Hence, a ChIP assay was performed using normal MAC-T cells. We initially designed six amplicons (P1, P2, P3, P4, P5 and P6) to determine whether menin binds to the promoter region of *Cyclin D1* (Fig. 5a, Supplementary Table S3). It was found that menin bound to the regions detectable by P1 (P < 0.001), P2 (P < 0.001), P3 (P = 0.02) and P4 (P < 0.001) (Fig. 5b). Based on the promoter sequence of *Cyclin D1*, up to six TATA-alike boxes and a CCAAT box exist within the P1 region, and a classical GC box with the conserved sequence GGGCGG exists in the P2 region. Thus, menin, known as a scaffold protein directly interacting with a number of different transcription factors [6], may regulate *Cyclin D1* transcription, by associating with the RNA polymerase II (Pol II) complex, binding to the promoter region of *Cyclin D1* containing TATA boxes, a CCAAT box and GC box elements near the transcription start site. Therefore, menin might regulate the G1/S transition of bovine mammary gland epithelial cells by binding to the *Cyclin D1* promoter.

### Menin is Abundantly Expressed in Mammary Epithelial Cells

**Fig. 6:**
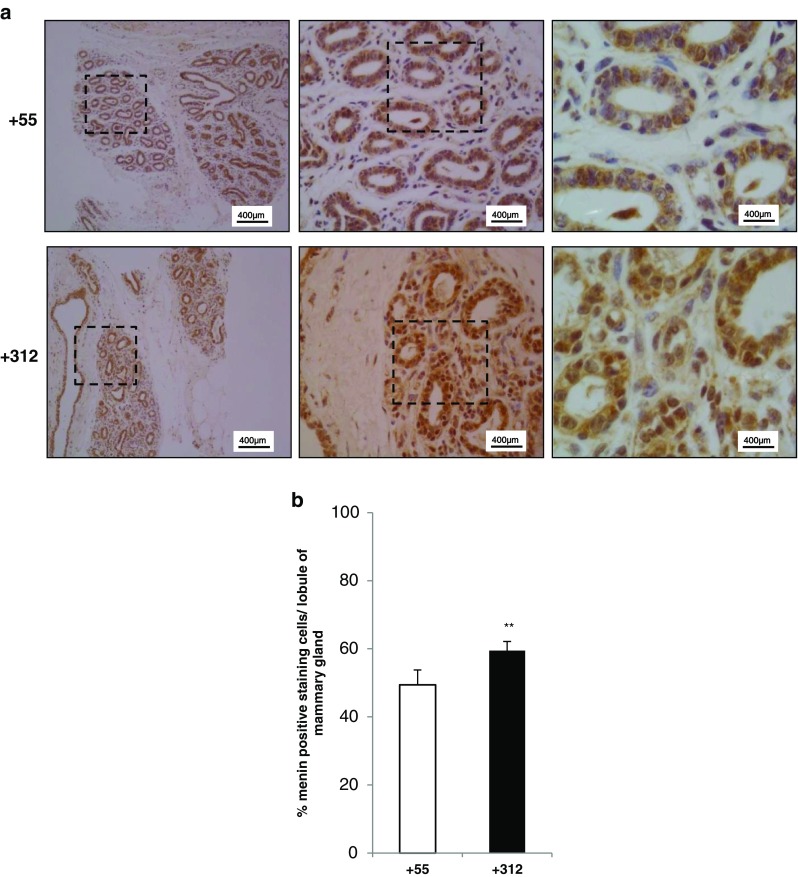
**Menin expression in mammary gland tissue of late lactation was more abundant than peak lactation staged tissue**. Mammary gland sections from + 55 days in milk (DIM) at the peak lactation stage and + 312 DIM in late lactation cows were assessed for menin expression by immunohistochemistry staining with anti-menin antibody. Normal rabbit IgG was used as a negative control. Representative results are shown in (**a**), bar = 200 µm. Brown indicates positive menin staining (brown in the nucleus and cytoplasm). The right panel is a high-powered magnification of the black dashed area in the left panel, with magnification of × 100, × 400, and × 1000, respectively. (**b**) Quantification of the averaged percentages of menin-expressed epithelial cells in randomly selected microscopic views from Figure A. Different numbers of microscopic views were randomly selected to quantify the mean density of positive staining signals in mammary epithelial cells at peak lactation stage (+ 55, n = 9) and late lactation stage (+ 312; n = 12), respectively. ** P < 0.01

To investigate whether menin is expressesed in epithelial cells in vivo, peak (+ 55) and late (+ 312) milking-stage mammary glands from non-pregnant dairy cows were stained using antibody against menin for immunohistochemistry analysis. Menin expression was detected in both the luminal and basal (myoepithelial) cell layers of the mammary epithelium (Fig. 6a), with much more abundant staining being observed in tissues from late lactation (+ 312) mammary glands than that in peak milk-stage (+ 55) mammary glands (Fig. 6b, P < 0.001).

## Discussion

### Regulation of the Expression of *Cyclin D1* by Menin at G1/S Phase Can Alter the Cell Cycle and Proliferation of Mammary Epithelial Cells

The *MEN1* gene encodes menin, a protein that is primarily nuclearly localized and has been shown to interact with a variety of transcriptional factors and other proteins [22, 24−24], suggesting that menin may act as an adaptor protein involved in the regulation of gene expression and, in particular, menin plays a complex role in cell cycle regulation [25]. It has recently been shown that menin mediates the repression of *Cyclin B2* expression through physical binding to its promoter and therefore inhibits the G2/M transition and cell proliferation [26]. Moreover, *MEN1*/menin was previously reported to participate in the regulation of cell cycle progression at G1/S phase [27, 30−30]. Progression through the cell cycle depends on the activity of cyclins (such as Cyclin D1) and/or cyclin-dependent kinase (CDK) inhibitors (such as p18, p21 and p27 proteins), which can cause G1 arrest and termination of DNA synthesis in S-phase [31, 32]. In quiescent rat pituitary somatolactotrope GH4C1 cells, menin blocks the progression from G0/G1 phase to S phase by regulating the expression of the CDK inhibitors p21 and p27 [33]. Indeed, menin has been shown to directly associate with the promoters of *p27* and *p18* and promotes the methylation of lysine 4 in histone H3 (H3K4) and the expression of these genes [34]. Additionally, menin has been shown to repress the activator of S-phase kinase (ASK)-induced cell proliferation, preventing cells from entering S phase [25, 29, 30]. Thus, menin may play an important regulatory role at G1/S phase by interacting with promoter regions and certain proteins that function as transcription factors.

In this study, we identified a cell cycle progression regulatory role for menin in mammary epithelial cells. Decreased expression levels of menin caused mammary epithelial cell growth arrest at G1/S phase as its physical binding to the promoter region of *Cyclin D1*, a crucial ligand protein of CDK4/CDK6 enables cell progression from G1 to S phase [28, 35]. In the promoter region of the bovine *Cyclin D1* gene where menin potentially binds, there are up to six TATA-like boxes and CCAAT box in the P1 region and a classical GC box with the conserved sequence GGGCGG in the P2 region, which are the preferred promoter elements where RNA polymerase II (Pol II) usually binds. Thus, a decrease in menin may inhibit the binding of Pol II and/or other transcription factors to the promoter, resulting in repression of *Cyclin D1* expression. Indeed, accompanying the Cyclin D1 repression observed under reduced menin expression, down-regulation of histone H3 lysine 4 trimethylation (H3K4me3; Supplementary Fig. S2) was found. Simultaneously, the up-regulation of *p18* (Supplementary Fig. S3) was found to be associated with lower menin expression, although the detailed underlying mechanisms are not yet clear.

### Abundant, but Modulated Expression of Menin in the Mammary Epithelium During Different Lactation Stages Indicates a Potential Regulatory Role of Menin in Epithelial Cell Survival

**Fig. 7:**
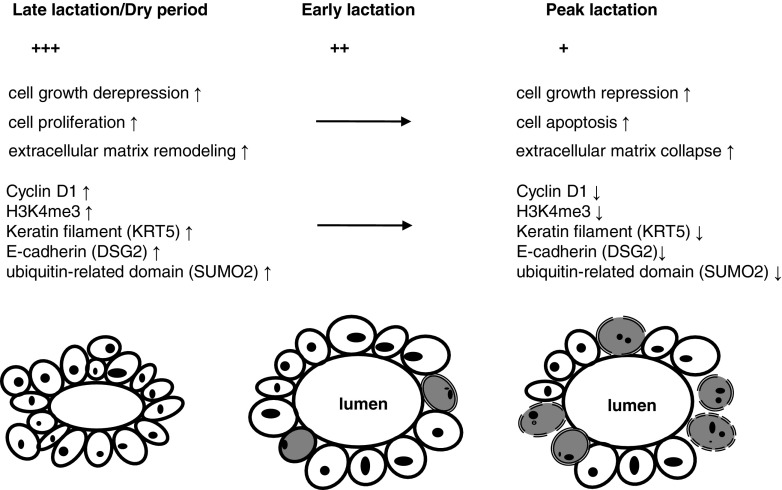
**Hypothetical model of the menin-mediated effect on mammary epithelial cell numbers and the lactation curve pattern of mammary glands**. The gradual decrease in menin expression after the initiation of lactation in mammary glands causes epithelial cell growth arrest in G1 stage through repression of *Cyclin D1*, also inducing cell apoptosis. Menin expression continues to decrease until reaching its lowest point at the peak lactation stage. However, in the late lactation stage and/or dry period (no lactation), menin expression levels are recovered, possibly related to differences in hormone secretion (Li et al. 2017; Karnik et al. 2007). The growth of epithelial cells becomes derepressed, and extracellular matrix degradation resulting from decreased menin ceases. Epithelial cells proliferate again, and the extracellular matrix undergoes remodeling, in preparation for the next lactation cycle. “+” indicates relative menin expression levels; grey-filled circles indicate G1-arrested mammary epithelial cells. Upward arrows indicate increased expression or activity, while downward arrows indicate decreased expression or activity

Menin was found to be abundantly expressed in the cell layers of the mammary epithelium during different stages of lactation, including the dry period (Fig. 6), indicating its potential regulatory role in epithelial cell survival. Notably, menin expression was slowly suppressed with advancing lactation and reached a lower point around the peak milk stage (supplementary Fig. S1, Fig. 7). However, by the involution stage, menin expression was slowly increasing. Provided that a single mammary gland from an individual animal was used to track the expression of *MEN1*/menin throughout the lactation period, a statistically significant change would be observed. More importantly, the expression model of menin throughout the lactation cycle in mammary glands was found to be negatively correlated with the normal lactation curve, showing that the milk yield continues to increase until peak lactation on days of 60–90, but decline thereafter until involution and/or the dry period.

The number of secretory mammary epithelial cells inside the mammary gland ultimately determines the potential milk yield of an animal. The mammary gland undergoes dramatic functional and metabolic changes during the milking period. For example, the number of epithelial cells slowly declines after lactation begins [15, 36]. The internal molecular regulatory mechanism of this phenomenon has long been a biological conundrum for the mammary biologist. Here, we found that *MEN1*/menin is the factor that controls the dynamic balance between cell proliferation and cell death in the lactating mammary gland, thus modulating the lactation curve pattern of the mammary gland (Fig. 7).

The decreasing expression of menin after lactation begins may slowly result in growth arrest of mammary epithelial cells through the cell cycle regulator *Cyclin D1* and even apoptosis, suggesting that *MEN1*/menin is one of the internal regulatory molecular mechanisms causing the decline in the number of epithelial cells. Indeed, gene expression profiling of bovine mammary glands suggested that the onset of lactation was accompanied by up-regulation of genes involved in milk synthesis, and by inhibition of genes related to cell proliferation [15, 36, 37]. Cyclin D1 was also found to be significantly down-regulated in early lactation mammary gland tissue compared to late pregnancy tissue (dry stage) [36, 38].

More specifically, with decreasing menin expression, mammary epithelial cells exhibited increased early apoptosis (Fig. 3c, Supplementary Figure S4) and extracellular matrix remodeling activity (Fig. 2e). Genes showing inhibited expression that involved in extracellular matrix activity included extracellular matrix components (keratin filament *KRT5*, laminin filament *LAMB3*), cell adhesion molecule (cadherin *DSG2*), proteolysis and catabolic processes (ubiquitin-related domain, *SUMO2*; oxidoreductase, *BLVRA*; hydrolase-like, *MEST*; peptidase, *ECE1*) and calcium ion binding (S100 calcium-binding protein A2, *S100A2*) (Fig. 2c). Interestingly, the role of menin in regulating cell–cell adhesion was also observed in mice with *Men1* inactivation-induced lesions in the mammary glands [13] and pancreatic islets [8, 39], although the underlying mechanism requires further investigation. Additionally, and of greater interest, similar groups of gene have been shown to be significantly suppressed in early lactation mammary glands compared with the dry period before parturition according to previous reports [36, 40]. A study in mice found that the communication between mammary epithelial cells and their environment became weak during the lactation period [41]. These results demonstrated that in the period before peak milk, the communications between epithelial cells and their extracellular matrix were slowly weakened [17, 18, 36, 37, 42], with increasing apoptosis of mammary epithelial cells and collapse of extracellular matrix (Fig. 7); these changes then slowly recover in the dry period because of the expression restoration of *MEN1*/menin, in preparation for the next lactation cycle. Thus, menin is at least one of the internal regulators inside the mammary gland that modulates epithelial cell numbers through the lactation cycle, although other factors are yet to be revealed.

Regarding the modulation of menin expression in mammary epithelial cells at different lactation stages, the reproductive hormone prolactin could be the upstream controller, which inhibits *MEN1*/menin expression [4, 12, 43]. In bovine mammary gland tissue, the different expression levels of menin during the lactation period may be a consequence of menin responding to the waves of reproductive hormone secretion, thus modulating mammary epithelial cell numbers. In addition to the role of menin in the control of normal mammary cell growth and proliferation, we found that menin can mediate the role of surrounding hormones and energy in milk protein synthesis in epithelial cells through the PI3K/Akt/mTOR pathway [12].

Considering these data together, we hypothesize that *MEN1*/menin could be one of the primary mediators controlling the growth of epithelial cells through the cell cycle progression factor Cyclin D1, dynamically modulating the lactation persistency and the lactation curve of normal mammary glands. The discovery of this unknown role of menin may shed light on the molecular mechanisms underlying the long-term biological conundrum of declining milk yield after peak lactation in mammary glands. There could be other factors besides of *MEN1*/menin playing similar regulatory role in mammary glands. This discovery would result in new ideas for adjusting animal milk production and a possible new mechanism in mammary biology.

## Materials and Methods

### Ethics Statement

All experiments were carried out according to the Regulations for the Administration of Affairs Concerning Experimental Animals published by the Ministry of Science and Technology, China (2004) and were approved by the Animal Care and Use Committee of Shandong Agricultural University, Shandong, China.

### Animals and Mammary Gland Tissue Collection

Fifteen healthy multiparous Holstein cows were biopsied for the mammary gland samples at Holstein Cattle Association Jiabao Farm in Shandong province. These cows were not pregnant, with an average parity of 2.67 ± 2.06 and an average weight of 602 ± 19.2 kg. Samples were obtained on lactation days (calving day as day 1) of 4.6 ± 1.5, 55 ± 4.3, 163 ± 6.24, 312 ± 24.6 and during the dry period (36 ± 6.8 days after milking stopped), representing 5 different lactation stages (3 animals for each stage) with a variable developmental status of their mammary glands. Mammary biopsies were collected using the Bard Magnum biopsy system (Bard Peripheral Vascular, Inc., Tempe, AZ, US) as described [12]. The mammary tissue samples were immediately frozen in liquid nitrogen and stored at − 80 °C for subsequent analysis or fixed in a 4% formaldehyde solution for immunohistochemistry. Cows were housed in a free stall barn with constant access to water and feed. Diets were formulated to meet all NRC (2001) recommendations for early- lactation, mid-lactation and/or dry cows using the Cornell-Penn-Miner system (CPM-Dairy, version 3.0.7) to meet the metabolizable energy and protein requirements. Feed was provided ad libitum, and the lactation cows were milked three times daily.

### Cell Culture and Transient Transfection Assays

Bovine mammary epithelial cells (MAC-T) were grown in Dulbecco’s modified Eagle’s medium (DMEM), containing 10% fetal bovine serum (FBS), penicillin (100 U/L) and streptomycin (100 mg/L) in a 5% CO_2_ atmosphere at 37 °C. The cells (5 × 10^5^ cells/well) were pre-seeded in 6-well culture plates in medium without antibiotics at a density of 70–80% confluence at 24 h prior to transfection.

siRNAs specific for the bovine *MEN1* gene were designed and synthesized (Ribobio, Guangzhou, China; Supplementary Table S1) and used for *MEN1*/menin knockdown. MAC-T cells were transfected with 50 nM target-specific pool siRNAs per well using OPTI-MEM® I Medium (Invitrogen, Carlsbad, CA, USA) and Lipofectamine 2000 (Invitrogen, Carlsbad, CA, USA) according to the manufacturer’s protocol, and a non-specific scramble negative control siRNA (Ribobio, Guangzhou, China) transfections were conducted at the same time. The cells were processed for protein and/or RNA isolation at 24 h post-transfection. All of the experiments were performed at least three times for each transfection. Preliminary experiments were conducted under the same condition by using separate and/or combinations of target siRNAs, so as to obtain the best knockdown efficiency.

### RNA Isolation and Quantitative Real-Time RT-PCR

Total RNAs were extracted from mammary tissues (about 20 ng) or MAC-T cells (5 × 10^5^ cells) using an RNA extraction kit (Tiangen Biotech, Beijing, China), followed by treatment with DNaseI (Ambion, Austin, Texas, USA). The quality of total RNA was assessed through agarose gel electrophoresis and the calculation of OD_260_/OD_280_. Oligo(dT)-primed first-strand cDNA was employed for quantitative real-time RT-PCR (qRT-PCR) using SYBR-Premix Ex Taq II (TaKaRa, Dalian, China) and an Mx3000p cycler (Stratagene, La Jolla, CA, USA). Each CT value was obtained from the averaged CTs of triplicate reactions. The expression level of target gene was normalized to its corresponding internal control β-actin. The data were plotted as fold change over their corresponding controls in MAC-T cells or certain lactation stage in mammary gland tissues. The 2^− ΔΔCT^ method was used to calculate the relative abundance of mRNA. All primer sequences are listed in Supplementary Table S2.

### Western Blotting

Total protein were extracted in RIPA lysis buffer (containing 1% PMSF) (Beyotime, Nanjing, China) from MAC-T cells after 24 h of transfection or mammary gland tissues. The proteins (approximately 20 μg of total protein) were separated on a 10% SDS–PAGE gel and transferred onto nitrocellulose membranes using 200 mA of constant current. The western blot was performed as per standard protocols. Primary antibodies against bovine menin (Bethyl Laboratories, Texas, USA), bovine cyclin D1 (Beyotime, Nanjing, China), and β-actin (Beyotime, Nanjing, China) were used at 1:1000 dilution. β-actin was used as the total protein loading control. The HRP-conjugated secondary antibody (Beyotime, Jiangsu, China) was diluted by 1000 as the working solution. Chemiluminescence detection was performed using BeyoECL Plus (Beyotime, Beijing, China). The data are shown as the expression level normalized to their corresponding negative controls. The results are representative of three independent experiments that were used to determine the statistical significance.

### High-Throughput Sequencing

RNAs from three independent *MEN1*-specific siRNA treatments of MAC-T cells collected at 24 h after transfection were extracted and pooled in equal proportions for SE50 high-throughput sequencing to identify the differentially expressed genes (DEG), as were their negative control siRNA treatments. mRNA Sequencing was performed on the Illumina HiSeq 2500 platform. Sequencing libraries were generated using the NEBNext® Ultra™ RNA Library Prep Kit for Illumina® (NEB, USA) following the manufacturer’s recommendations and index codes were added to attribute the sequences to each sample, followed by assessment of library quality using the Agilent Bioanalyzer 2100 system. Raw data (raw reads) in the fastq format were first processed with in-house perl scripts. All downstream analyses were based on high quality clean reads of 10 M. The index for the reference genome was built using Bowtie v2.0.6, and paired-end clean reads were aligned to the reference genome using TopHat v2.0.9. Prior to differential gene expression analysis, for each sequenced library, the read counts were normalized and standardized with the edgeR program package using the method of trimmed mean of M values (TMM). Differential expression analyses of two conditions were performed using the DEGSeq R package (1.12.0). The *P* values were adjusted via the Benjamini and Hochberg method. A corrected *P*-value of 1.3E-5, *q*-value of 0.001 and log2 (fold-change) of 0.3 were set as the threshold for significantly differential expression. Gene Ontology (GO) enrichment analysis of differentially expressed genes was implemented with the GOseq R package, in which gene length bias was corrected. GO terms with corrected *P*-values of less than 0.05 were considered significantly enriched with differentially expressed genes.

### Cell Proliferation and Cell Cycle Analysis

Equal number of MAC-T cells (1 × 10^6^) were transfected with *MEN1*-specific siRNAs for 24 h as well as the corresponding negative control. The same amount of cells (2.5 × 10^5^) were harvested, fixed and analyzed in Vindelov’s propidium iodide buffer for analysis of the distribution of cell cycle phases (FACSCalibur, BD Biosciences). All of the experiments were performed three times for each transfection.

### Cell Apoptosis Assays

Apoptosis levels were detected in *MEN1*-specific siRNA-treated MAC-T cells (2.5 × 10^5^) at 24 h post-transfection, as well as the untreated and scramble siRNA transfected cells, using an annexin V-FITC Apoptosis kit (Biovision) for immunofluorescence analysis, followed by cell analysis via flow cytometry (FACSCalibur, BD Biosciences) employing Cell Quest Pro software. Aliquots of the same cells (about 5 × 10^3^) were also stained with annexin V (green) and propidium iodide (red) and mounted in DAPI (blue)-containing anti-fade mounting medium. Microscopy and photomicrography were performed with AxioObserver Z1 (Zeiss, Thornwood, NY, USA).

### Chromatin Immunoprecipitation (ChIP)

Briefly, 5 × 10^6^ normal MAC-T cells were subjected to cross-linking with 1% formaldehyde, after which the cells were lysed in 1 ml of SDS lysis buffer and sonicated with a Bioruptor sonicator (UCD-200TM-EX, Diagenode, Belgium) to shear the chromatin into 200 – 1,000 bp fragments. The sonicated cell lysates (100 μl of the 1 ml supernatant) and 2 μg of antibodies were used for each immunoprecipitation. Anti-menin (Bethyl Laboratories, Montgomery, TX, USA) and anti-rabbit IgG (Sangon Biotech, Shanghai, China) were used for ChIP analyses. The precipitated DNA was used as template for quantitative real-time PCR using the SYBR-Premix Ex Taq II (TaKaRa, Dalian, China). Primer pairs screening of 1200 bp promoter region of *Cyclin D1* gene were designed and synthesized, their detailed information were listed in Supplementary Table S3. For quantitative real-time PCR, CT values for each ChIP were obtained from triplicate reactions. The results were representative of three independent ChIP experiments, showing as the percentage of input by quantifying the amount of chromatin obtained from immunoprecipitation relative to the amount in the input samples.

### Immunohistochemistry

Tissues dissected from the mammary glands of cows were fixed in 4% paraformaldehyde and embedded in paraffin. Sections (5 mm thickness) of the tissues were then subjected to standard immunohistochemistry staining. Detection of menin was performed incubating the slides with a rabbit polyclonal antibody (1:100; Bethyl Laboratories, Texas, USA) for 2 h at 37 °C. After the slides were washed, they were treated with HRP-conjugated goat anti-rabbit IgG (1:50; Beyotime, Nanjing, China) for 1 h at 37 °C. Images were captured using a microscope (Leica TCS SP2 AOBS, Germany). Image- Pro Plus (IPP) 6.0 software was used to quantify the mean density of positive-menin signals in mammary epithelial cells by randomly selecting different numbers of microscopic views.

### Statistical Analysis

The data are presented as the mean ± S.E.M. of at least three independent experiments. Statistic differences among groups were compared with one-way ANOVA and difference between pair-designed experiments were compared with Student’s *t*-tests by using SAS8.2 software (SAS Institute Inc., Cary, USA). Significant differences were declared when *P-*values were < 0.05 (*) or < 0.01 (**).

## Electronic supplementary material

Below is the link to the electronic supplementary material.

Supplementary material 1 (PDF 344 KB)
