# Supplementary material for: Menin Modulates Mammary Epithelial Cell Numbers in Bovine Mammary Glands Through Cyclin D1
Source: J Mammary Gland Biol Neoplasia. 2017 Nov 29;22(4):221–33. doi: 10.1007/s10911-017-9385-8 (PMC5854757; doi:10.1007/s10911-017-9385-8)
Supplement: Supplementary file 1 — Supplementary material 1 (PDF 344 KB) [file 10911_2017_9385_MOESM1_ESM.pdf]

Menin modulates mammary epithelial cell numbers in bovine mammary glands through *Cyclin D1*

Kerong Shi[*], Xue Liu, Honghui Li, Xueyan Lin, Zhengui Yan, Qiaoqiao Cao, Meng Zhao, Zhongjin Xu, and Zhonghua Wang[*]

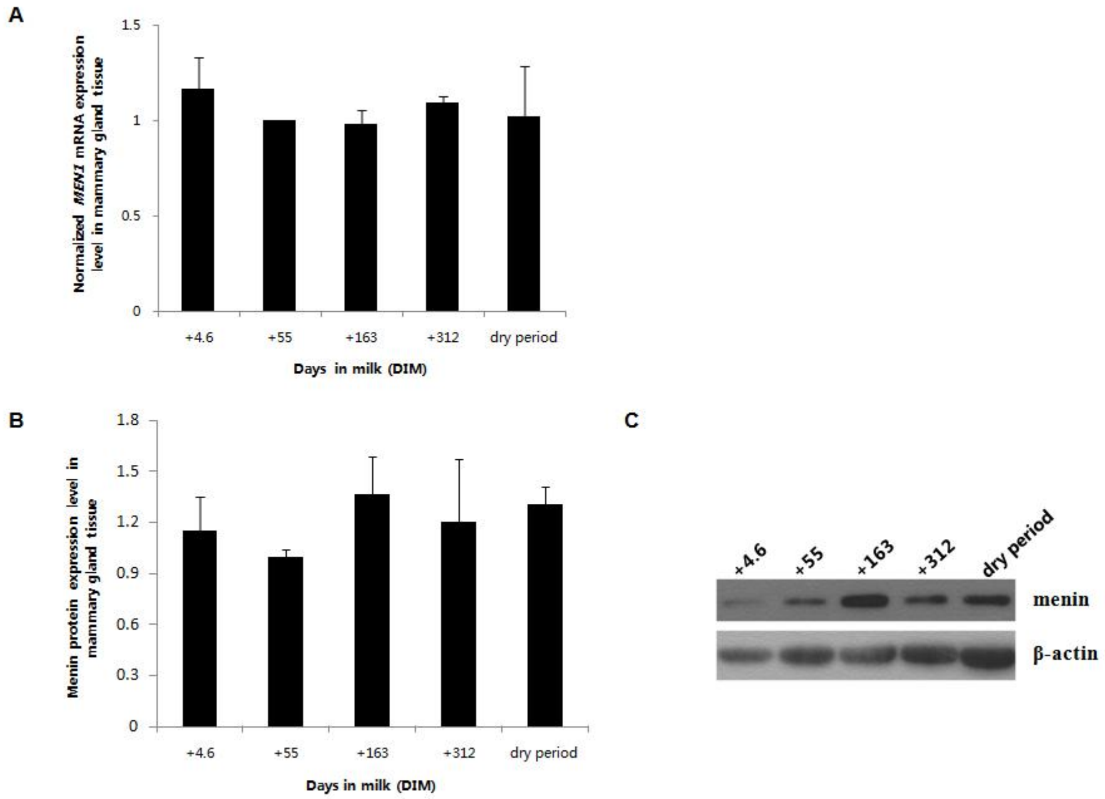

Supplementary Fig. S1. *MEN1*/menin expression in mammary gland tissue decreases in the early lactation period and increases by the end of lactation and in the dry period. To investigate the expression levels of *MEN1*/menin during lactation cycle in the mammary glands of dairy cows, mammary gland tissues from 5 different lactation stages of cows on days in milk (DIM) + (4.6±1.5), +(55±4.3), +(163±6.24) and +(312±24.6) as well as during the dry period, were used to analyze the expression levels of *MEN1* mRNA (A) and its encoded protein menin (B) via qRT-PCR and western blotting. The data are shown as relative expression levels normalized to an internal control β-Actin. The expression level of *MEN1*/menin at each stage was determined in two or three different individuals at each lactation stage. The results showed that the levels of both *MEN1* mRNA (A) and the menin protein (B) slowly decreased with advancing lactation, but later increased after the peak milking stage through the dry period (or involution stage), with the lowest expression level of menin being observed around the peak lactation stage (55±4.3 days in milk, DIM). One-way ANOVA analysis indicated no statistical difference among different lactation

stages (*P*=0.108 and *P*=0.114 for mRNA and protein expression, respectively.) (C) Representative western blot images of the expression of menin and the loading control β-actin are shown.

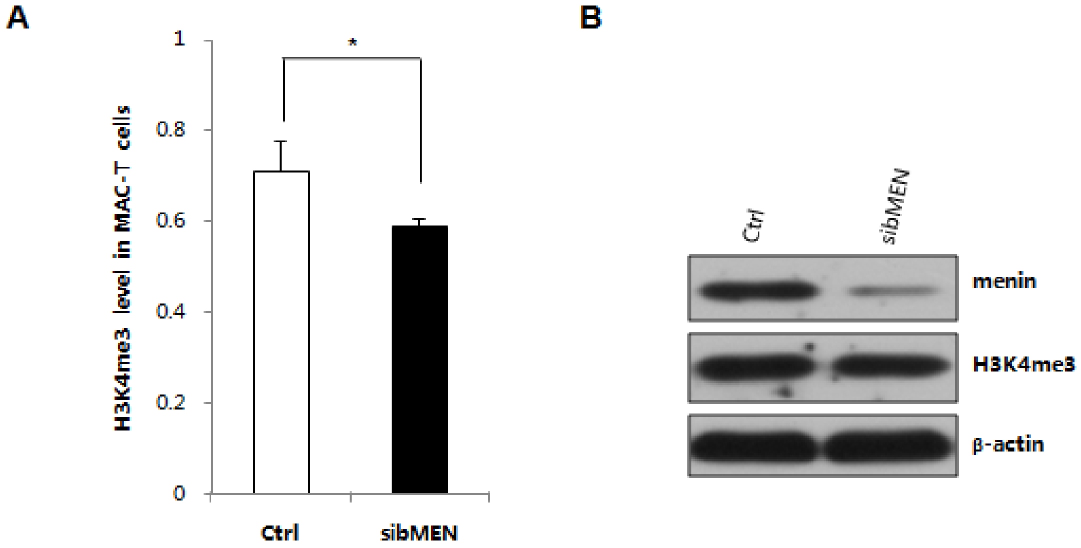

Supplementary Fig. S2 Besides *CycinD1* expression suppression, histone H3 lysine 4 trimethylation (H3K4me3) was inhibited upon *MEN1*/menin knockdown in mammary epithelial cells. (A) Histone H3 lysine 4 trimethylation level was decreased in MAC-T cells at 24h after transfected with MEN1-specific siRNAs (sibMEN), compared with its negative control (Ctrl) (n=3). * $P<0.05$. (C) Representative western blot images using antibodies against-menin (Bethyl), anti-H3K4me3 (Sangon) and anti-β-Actin (Beytime) are shown. β-Actin serves as a lysate loading control.

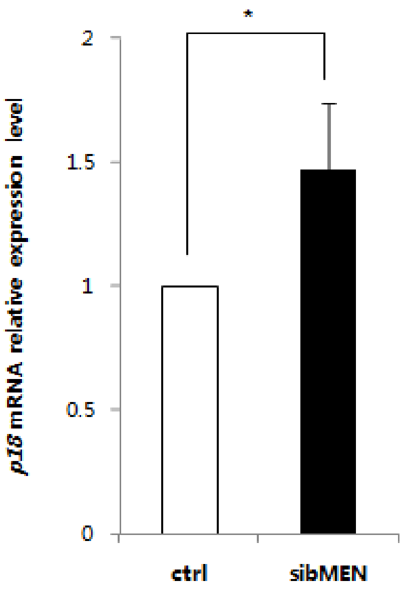

Supplementary Fig. S3 Cell progression negative regulator gene *p18* was up-regulated in mammary epithelial cells. Expression of *p18* was assessed at 24h in the MAC-T cells transfected with *MEN1*-specific siRNAs (sibMEN), as well as its negative control (Ctrl). Data are shown as relative expression levels normalized to an internal control β-Actin. ***P*<0.05.

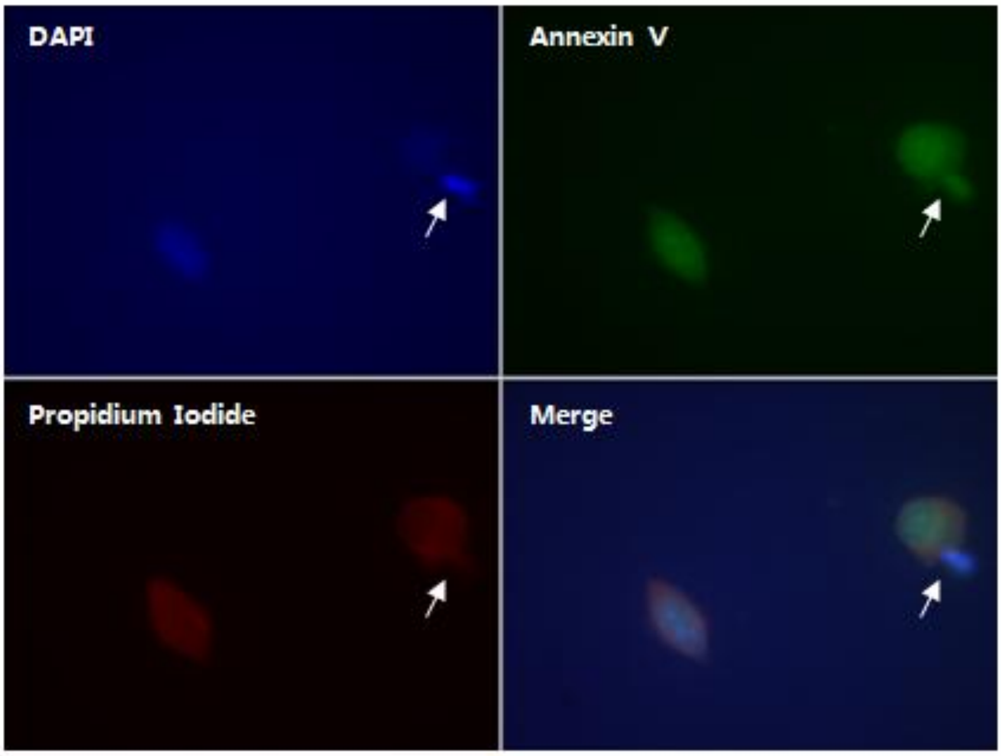

Supplementary Fig. S4 Suppression of *MEN1*/menin expression induced mammary epithelial cell apoptosis. Mammary epithelial cells MAC-T were exposed to *MEN1*-specific siRNAs for 24 hours and assessed for the cell apoptosis by Annexin V/propidium iodide (PI) staining. Cells were stained with Annexin V (green) and PI (red) and mounted in DAPI-containing anti-fade mounting medium. The apoptotic cells with broken cell membrane can be stained by both Annexin- V-FITC and propidium iodide. The two epithelial cells in the images showed apoptosis, with positive for both annexin V and PI staining. Arrow shows the cell in late apoptosis, with broken cell membrane and fragmented nuclear DNA waded out.

Supplementary Table S1 Sequences information of siRNAs specific to *MEN1* gene[1,2]

**Name**	**Sense(5’-3’)**	**Anti-sense (3’-5’)**
sibMEN-1	CCGUUGACCUGUCUCUCUA dTdT	dTdT GGCAACUGGACAGAGAGAU
sibMEN-2	CCACUGUCGUAACCGCAAU dTdT	CCACUGUCGUAACCGCAAU dTdT
sibMEN-3	CCGAGUGACUACACGCUUU dTdT	dTdT GGCUCACUGAUGUGCGAAA

[1]The “dT” letters denote deoxythymines.

[2]A scramble siRNA was also used in the study as the negative control. We can't provide its sequence because it belongs to commercial confidentiality of Ribobio (Guangzhou, China).

Supplementary Table S2 Primer sequences of target genes detected for expression analysis in MAC-T cells and mammary gland tissues

Gene	Primers	Primer sequences (5’→3’)	Product size
MEN1	Forward	GATGGAGGTGGCATTTATGG	256bp
	Reverse	GATGTGCTCATCCCGGTAGT	
β-actin	Forward	CCCAGCACAATGAAGATCAA	180bp
	Reverse	TAGAAGCATTTGCGGTGGAC	
CyclinD1	Forward	AGAAGTGCGAGGAAGAGGT	186bp
	Reverse	CGGATGGAGTTGTCAGTGT	
CyclinD3	Forward	AGCGCTGTGAAGAGGAAGTC	224bp
	Reverse	CAAGACCAGCACCCAAT	
CyclinB1	Forward	AAAGGCTCCTGTACCTGTGC	215bp
	Reverse	CTCCGTCTTCTGCATCCACA	
CyclinB2	Forward	TTCTCCCACACCTCAGGACA	276bp
	Reverse	AGCAGCCTGAACTTGGAGTG	
CyclinA2	Forward	AGGACCAAGAAAGCCACTGG	292bp
	Reverse	CAGGGTCTCGTTCTGCAGTT	
CDK1	Forward	CAGCTGGCTACTCAACTCCA	162bp
	Reverse	CCACTTCTGGCCACACTTCA	
CDK4	Forward	GGAGACCAAAGTGACCCTGG	212bp
	Reverse	GCTTGACTGTCCCACCACTT	
CDK6	Forward	CGAGGCCTGGACTTTCTTCA	226bp
	Reverse	CAACGCTCCAGAGATCCACA	
p18	Forward	CATTCATGATGCGGCCAGAG	288bp
	Reverse	CATTGCAGATTCGCAGCTCC	

Supplementary Table S3 Primer information of the six amplicons of the promoter region of *Cyclin D1* gene used in the ChIP assay

Name	Promoter region [1]	Primer sequences (5'→3')	Product size (bp)
P1	-1342 ~ -1103	F: TGTTAAGACTGGGTAAGATGTCC<br>R: TGAGCCTGAGGTGGTTGG	240
P2	-901 ~ -692	F: TGTTAAATTGCCGGCACAGG<br>R: TTTCAGCCTAACATGCGCTC	210
P3	-602 ~ -442	F: CCCTGAGTCCCTGAATGGTA<br>R: GGGTTCGGTGACACTCTGAG	160
P4	-374 ~ -223	F: TCTGCAGAGCCACCTTCAC<br>R: CGAAGCCGGGTTTTCATAGA	152
P5	-242 ~ -31	F: TCTATGAAAACCCGGCTTCG<br>R: AAACTCCCCTGTAGTCCGTG	212
P6	-19 ~ +194	F: GCAAAGTCCTAGAGCCTCCA<br>R: TCTCCATCTCGCAGCACAG	213

[1] shown is the start and end position of the target region. Given the transcription start site is +1, its upstream region is labeled as "-" using increasing number from 3' to 5', while, its downstream region labeled as "+".
